# Development of a prediction model for radiotherapy response among patients with head and neck squamous cell carcinoma based on the tumor immune microenvironment and hypoxia signature

**DOI:** 10.1002/cam4.4791

**Published:** 2022-05-03

**Authors:** Guang‐Li Zhu, Kai‐Bin Yang, Cheng Xu, Rui‐Jia Feng, Wen‐Fei Li, Jun Ma

**Affiliations:** ^1^ Department of Radiation Oncology Sun Yat‐sen University Cancer Center, State Key Laboratory of Oncology in South China, Collaborative Innovation Center for Cancer Medicine, Guangdong Key Laboratory of Nasopharyngeal Carcinoma Diagnosis and Therapy Guangzhou P. R. China

**Keywords:** gene signature, head and neck squamous cell carcinoma, hypoxia, immune, radiosensitivity

## Abstract

**Introduction:**

The immune system and hypoxia are major factors influencing radiosensitivity in patients with different cancer types. This study aimed at developing a model to predict radiotherapy response in patients with head and neck squamous cell carcinoma (HNSCC) based on the tumor immune microenvironment and hypoxia signature.

**Materials and Methods:**

We first evaluated the hypoxia status and tumor immune microenvironment in the Cancer Genome Atlas (TCGA) cohort by using transcriptomic data. Differentially expressed genes (DEGs) were identified between the “high immunity and low hypoxia” and “low immunity and high hypoxia” groups and those DEGs significantly associated with disease‐specific survival in the univariate Cox regression analysis were selected as the prognostic DEGs. We selected the immune hypoxia–related genes (IHRGs) by intersecting prognostic DEGs with immune and hypoxia gene sets. We used the IHRGs to train a multivariate Cox regression model in the TCGA cohort, based on which we calculated the IHRG prognostic index (IHRGPI) for each patient and validated its efficacy in predicting radiotherapy response in the Gene Expression Omnibus cohorts. Furthermore, we explored potential mechanisms and effective combinational treatment strategies for different IHRGPI groups.

**Results:**

Five IHRGs were used to construct the IHRGPI, which was used to dichotomize the cohorts. The patients with lower IHRGPI showed a better radiotherapy response across different cohorts and endpoints, including overall survival, progression‐free survival, and recurrence‐free survival (*p* < 0.05). Patients with higher IHRGPI showed greater hypoxia and lesser immune cell infiltration. A lower IHRGPI indicated a better immunotherapy response, while a higher IHRGPI indicated a better chemotherapy response.

**Conclusions:**

IHRGPI is promising for predicting radiotherapy response and guiding combinational treatment strategies in patients with HNSCC.

## Graphical abstract

The immune hypoxia related genes prognostic index (IHRGPI), which was based on the tumor microenvironment and hypoxia signature, was developed and validated in patients with head and neck squamous cell carcinoma for prediction and identification of beneficiaries of radiotherapy. The patients with lower IHRGPI showed a better radiotherapy response across different cohorts and endpoints, including overall survival, progression‐free survival, and recurrence‐free survival, and the patients with higher IHRGPI showed greater hypoxia and lesser immune cell infiltration. A lower IHRGPI indicated a better immunotherapy response, while a higher IHRGPI indicated a better chemotherapy response.

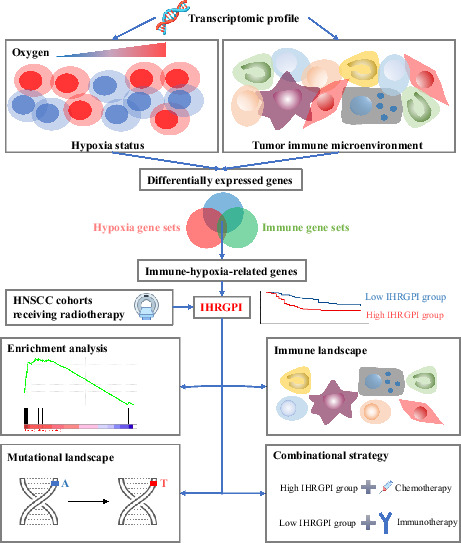

## 1 INTRODUCTION

Head and neck squamous cell carcinoma (HNSCC) is a heterogeneous group of tumors that originate from the mucosal epithelium of head and neck, including the lip, oral cavity, larynx, and pharynx (excluding the nasopharynx). It is the most common type of head and neck cancer and the sixth leading cancer worldwide. In 2018, its incidence and mortality were reported to be 890,000 and 450,000, respectively.^1^ However, hitherto, no effective screening method is available for HNSCC, and more than 50% of the cases were found to be locoregionally advanced (stage III–IV) at diagnosis. Surgery, radiotherapy, and chemotherapy are the major treatment modalities for non‐metastatic HNSCC. Surgery and radiotherapy are the mainstays in most cases. For high‐risk patients who have undergone resection, postoperative adjuvant radiotherapy alone or chemoradiotherapy is recommended. Considering individual differences in response to radiotherapy, the optimal criteria for treatment decisions remained unestablished. However, individual differences in radiosensitivity greatly limit the therapeutic effect of radiation. Therefore, current treatment plans might be inadequate for the management of radioresistant HNSCC. Furthermore, since radiotherapy has deleterious effects on adjacent normal organs and tissues, which might impair phonation or swallowing, it might not be the optimal choice for radioresistant HNSCC patients. Recently, radiotherapy techniques have improved greatly. The advent of intensity‐modulated radiotherapy (IMRT) has resulted in a better dose‐volume distribution and greatly reduced toxicities. However, compared with two‐dimensional radiotherapy or three‐dimensional conventional radiotherapy, IMRT did not achieve better recurrence‐free survival (RFS), disease‐free survival (DFS), and overall survival (OS).^2^ Under such circumstances, increasing the strength of combinatory treatment modalities or switching to other treatment modalities might be necessary. Thus, considering radiosensitivity during treatment decisions is necessary for choosing the optimal curative and safe approach.

In the traditional theory regarding the biological effects of irradiation, the factors affecting radiotherapy response are known as the “5 Rs”: Repair of radiation‐induced DNA damage, Redistribution/Reassortment of cell cycles, Regeneration/Repopulation of survived cells, Reoxygenation and cellular intrinsic Radiosensitivity.^3^ Oxygen is a radiosensitizer because it plays a key role in the indirect radiation effect, in which radiation induces free radical generation to attack the target molecules. Compared with euoxic cells, hypoxic cells are 2–3 times more resistant to radiation.^4^ With the rapid development of immunotherapy, the 6th R, Reactivation of the immune system, was proposed in 2019.^5^ Radiation attacks DNA and induces DNA damage repair, during which the tumor cells become more visible to the immune system, and the tumor microenvironment is altered. Innate and adaptive immunity is initiated to cause a specific immune response against the tumor and immunogenic cell death. Besides the local effect, the irradiated tumor becomes an in situ vaccine, inducing a systemic immune response. Non‐irradiated metastases also shrink under the attack of the immune system, which is known as the abscopal effect. The findings associated with the 6th R are changing our understanding of radiosensitivity, which was previously restricted to tumors themselves.

The development of a model to predict radiotherapy response among HNSCC patients could guide individualized treatment decisions and improve prognosis. With the rapid development of sequencing techniques, many gene signatures were developed to predict radiosensitivity.^6^, ^7^, ^8^, ^9^ However, most of them were purely statistically driven and not based on radiobiological theories^7^, ^8^, ^9^; therefore, the underlying mechanism is hard to explain. Furthermore, most did not consider the impact of the tumor immune microenvironment, because they were developed in cell lines.^6^ Hence, in this study, we tried to use transcriptomic data to develop and validate an immune hypoxia–related gene prognostic index (IHRGPI) based on the tumor immune microenvironment and hypoxia signature to predict radiotherapy response in HNSCC patients, investigate the underlying mechanisms, and explore potential effective combinatory treatment strategies.

## 2 MATERIALS AND METHODS

### 2.1 Data acquisition and preparation

**FIGURE 1:**
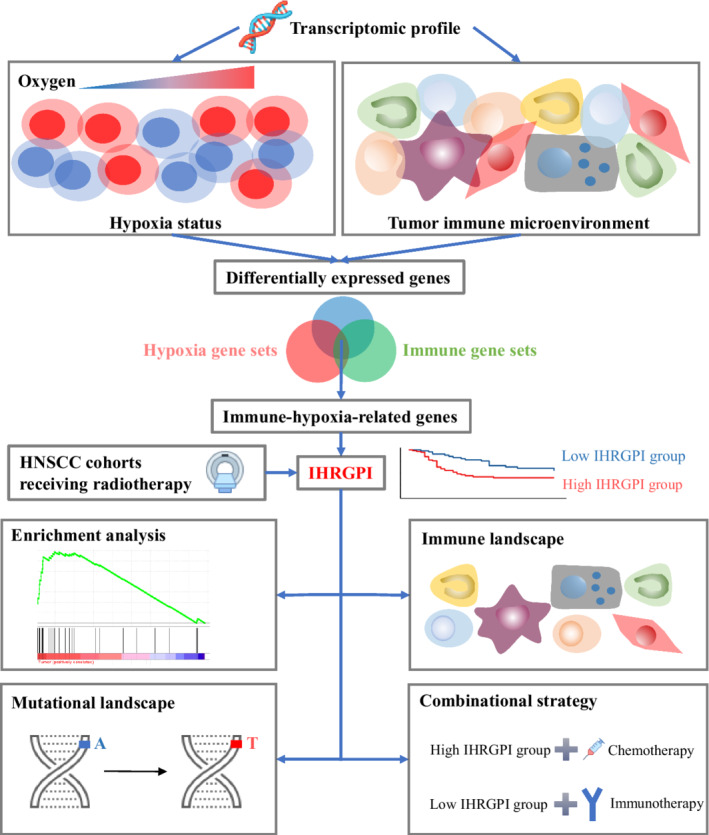
Schematic diagram of the study design. IHRGPI, immune‐hypoxia‐related gene prognostic index; HNSCC, head and neck squamous cell carcinoma

Figure 1 presents the workflow of this study. The Cancer Genome Atlas (TCGA) HNSCC cohort consists of 501 HNSCC patients, providing 546 HNSCC samples including 500 primary tumor samples, two metastatic tumor samples, and 44 para‐cancer samples. The cohort's RNA‐Seq data were downloaded from the TCGA database (https://portal.gdc.cancer.gov) by using the R package “TCGAbiolinks”^10^ on June 1, 2021. The corresponding clinicopathological information was downloaded from the cBioPortal database^11^, ^12^ (https://www.cbioportal.org/). The microarray gene expression data and clinicopathological information of two HNSCC cohorts were downloaded from Gene Expression Omnibus (GEO) database^13^ (accession numbers: GEO cohort‐1 and GEO cohort‐2, GSE117973 and GSE39366,^14^ respectively). Disease‐specific survival (DSS) and RFS were the only available endpoint for GEO cohort‐1 and cohort‐2 respectively. Therefore, DSS, which was available for both the TCGA cohort and the GEO cohort‐1, was chosen as the primary clinical endpoint. The other endpoints, including RFS, PFS, and OS data from GEO cohort‐2, GEO cohort‐1 and TCGA cohort, respectively, were used to investigate the model's generalizability for the prediction of the radiotherapy effect on the other endpoints. Primary tumor samples from patients who received radiotherapy and whose complete follow‐up data were available were included in the analysis. The study strictly followed the principles of the Declaration of Helsinki and data access policies of the TCGA and GEO database. The requirements of patients' informed consent and institutional review board approval were waived given the datasets were de‐identified and publicly available.

### 2.2 Identification of hypoxia and tumor microenvironment status

To investigate the patients' hypoxia status, the “WINTER” hypoxia gene set containing 95 hypoxia‐related genes upregulated in head and neck tumor samples^15^ was downloaded from the molecular signature database^16^, ^17^ (MSigDb version 7.4, https://www.gsea‐msigdb.org/gsea/msigdb/cards/WINTER_HYPOXIA_UP.html). The included TCGA samples were divided into two groups per the Euclidian distance of their expression patterns of the hypoxia gene sets, which were visualized by heatmap and t‐distributed Stochastic Neighbor Embedding (t‐SNE) with the R packages “pheatmap” and “Rtsne.” The group with the obviously upregulated hypoxia gene set was annotated as the “high hypoxia” group, while the other as the “low hypoxia” group. To validate the hypoxia status, the differences in the expression levels of the genes involved in the *HIF1* signal transduction pathway were compared between the two groups by using the limma algorithm.^18^ Genes with a false discovery rate (FDR) adjusted *P*‐value <0.05 and an absolute log2 (fold change) >1 were considered as differentially expressed genes (DEGs). The genes involved in the *HIF1* signal transduction pathway were downloaded from the Kyoto Encyclopedia of Genes and Genomes (KEGG) database^19^ (https://www.kegg.jp/; ID:04066). Finally, the difference in DSS between the two groups was evaluated using Kaplan–Meier curves with the log‐rank test.

To infer the patients' tumor microenvironment status, the Estimation of STromal and Immune cells in MAlignant Tumor tissues using Expression data (ESTIMATE)^20^ was applied for the TCGA cohort. Using ESTIMATE, the immune and stromal scores were calculated to estimate the degree of immune and stromal cell infiltration in tumor tissues based on the gene expression profile. The patients were divided according to the cutoff of the ESTIMATE immune score determined by maximally selected log‐rank statistics with the R package “survminer.” The group with the higher immune score was annotated as the “high immunity” group, while the other as the “low immunity” group. To explore differences in the tumor microenvironment between the two groups, the proportions of different types of tumor‐infiltrating cells from the transcriptomic data of each tumor sample were estimated using the Microenvironment Cell Populations‐counter (MCP‐counter) algorithm,^21^ which allows quantification of the absolute abundance of eight immune and two stromal cell populations and between‐sample comparison. Finally, the difference in DSS between the two groups was evaluated based on Kaplan–Meier curves with the log‐rank test.

### 2.3 Identification of IHR subtypes and their association with survival

Based on the findings obtained from the aforementioned steps, the TCGA cohort was further divided into three groups by using a two‐dimensional index combining the tumor microenvironment and hypoxia status: including “high immunity and low hypoxia”, “low immunity and high hypoxia”, and “mixed” groups. Finally, the difference in DSS between the “high immunity and low hypoxia” and “low immunity and high hypoxia” groups was evaluated using Kaplan–Meier curves with the log‐rank test.

### 2.4 IHRGPI development and validation

To build a prognostic model for radiotherapy response prediction in the TCGA cohort, first, the “limma” algorithm^18^ was used to identify DEGs between the “high immunity and low hypoxia” and “low immunity and high hypoxia” groups (absolute log2[fold change] > 1; FDR‐adjusted *P*‐value <0.05). Next, univariate Cox regression analysis was performed to select prognostic DEGs that were significantly associated with DSS (*P* < 0.05). The hallmark hypoxia gene set was retrieved from MSigDb, and an immune gene set was compiled by combining all immune‐related gene sets retrieved from InnateDB (http://www.innatedb.com). Prognostic DEGs that were present in both the immune and hallmark hypoxia gene sets were selected as the IHRGs for model training. To improve comparability affected by platform differences, the expression levels of the genes in the TCGA and GEO datasets were individually transformed to *z*‐scores.

A multivariate Cox regression model was trained with the above selected IHRGs in the TCGA cohort. IHRGPI was the sum of the products of the expression value of each gene for each patient and their coefficients in the trained multivariate Cox model. To investigate whether the IHRGPI score is an independent prognostic factor of radiotherapy response, univariate and multivariate Cox regression analyses incorporating common clinical variables and IHRGPI were performed for the TCGA cohort. According to IHRGPI with 0 as the cutoff value, patients in each cohort were dichotomized into radiosensitive (IHRGPI<0) and radioresistant (IHRGPI≥0) groups. GEO cohort‐1 served as an external validation source. To reduce the bias introduced by confounding factors, 1:1 propensity score matching was performed for T and N stages in the TCGA cohort and GEO cohort‐1 respectively. The prognostic power of the IHRGPI group in predicting radiotherapy response was evaluated using Kaplan–Meier curves with the log‐rank test and univariate Cox regression model in both TCGA and GEO cohorts. Furthermore, to explore the generalizability of IHRGPI to other endpoints, the prognostic significance was explored with OS, PFS, and RFS as an endpoint in the TCGA cohort, GEO cohort‐1, and GEO cohort‐2, respectively.

### 2.5 Comprehensive analysis of hypoxic, tumor‐microenvironmental, and molecular characteristics in the two IHRGPI groups

To illustrate the mechanism underlying the differences in radiotherapy response in the two IHRGPI groups, first, differential expression analysis was performed with the limma algorithm^18^ for both groups in the TCGA cohort to identify radiosensitivity‐associated DEGs (absolute log2[fold change] >1; FDR‐adjusted *P*‐value <0.05). The radioresistant group was set as the control group. Furthermore, functional enrichment analysis was performed with these DEGs by using the R package “clusterProfiler”.^22^ Radiosensitivity‐associated DEGs were first mapped to Gene Ontology (GO, http://geneontology.org/) terms and KEGG (https://www.genome.jp/kegg/) pathways. Next, the correlation between the genes adopted in the trained model was explored using GO terms. Finally, gene set enrichment analysis (GSEA) was performed to identify the enriched gene sets in both groups. The gene sets utilized in the GSEA were retrieved from MSigDb, including GO (C5) and Hallmark (H) gene sets. To analyze the difference in the tumor immune microenvironment between the two groups, CIBERSORT^23^ (https://cibersort.stanford.edu/) was used to estimate and compare the relative abundance of different types of immune or stromal cells from the gene expression profile. Regarding genetic mutational analyses, the somatic mutational data of the TCGA cohort were downloaded from TCGA database by using the R package “TCGAbiolinks”.^10^ Somatic mutations in the IHRGPI groups were analyzed and visualized, and the tumor mutational burden (TMB) was calculated using the R package “maftools”.^24^ TMB was defined as the total number of non‐synonymous somatic mutations per megabase (Mb) of the tumor genome. Finally, the ChIP‐X Enrichment Analysis 3 (ChEA3) tool^25^ was used to perform transcription factor enrichment analysis to identify transcription factors significantly associated with the selected IHRGs.

### 2.6 Exploration of treatment strategies for two IHRGPI groups

To explore the potential effective combinational strategies of radiotherapy and immunotherapy, the Tumor Immune Dysfunction and Exclusion (TIDE) altorithm^26^ (http://tide.dfci.harvard.edu) was used to predict the patients' response to immune checkpoint inhibitors. Tumors with higher levels of cytotoxic T cells (CTLs) achieved immune escape by inhibiting CTL functions, while tumors with lower levels of CTLs achieved immune escape by inhibiting CTL infiltration. The TIDE score takes both conditions into consideration to evaluate the potential of tumor immune escape based on gene expression profiles. A higher TIDE score indicates a higher immune evasion phenotype and worse response to immune‐checkpoint inhibitors. Next, the expression levels of several well‐studied targets for immune checkpoint inhibitors were compared, including *CTLA4*, *PD1*, *PDL1*, *B7H3*, *B7H4*, *B7H5* (*VISTA*), *BTLA*, *LAG3*, *TIM3*, *TIGIT*, *PVRIG*, *A2aR*, *CD73*, and *NKG2A* ^27^, ^28^, between the two groups.

To explore the potential effective combination strategies of radiotherapy and chemotherapy, the R package “pRRophetic” was used to predict individual sensitivity to three commonly used chemotherapeutic drugs for HNSCC: cisplatin, gemcitabine, and docetaxel. The “pRRophetic” algorithm^29^ was developed based on the public pharmacogenomics database Genomics of Drug Sensitivity in Cancer (https://www.cancerrxgene.org). It built a ridge regression model from gene expression profile and drug sensitivity data.

### 2.7 Statistical analysis

Statistical analysis was performed using the R software (v.4.0.1, https://www.r‐project.org/about.html). The independent Student's *t*‐test was used to compare differences in continuous variables following normal distribution between two groups, while the Wilcoxon test was performed for continuous variables that did not follow normal distribution. The chi‐square test or Fisher's exact test was used to test differences in categorical variables between two groups. Multiple testing was adjusted by the FDR method.

## 3 RESULTS

### 3.1 Hypoxia status and immunophenotype of the TCGA cohort

**FIGURE 2:**
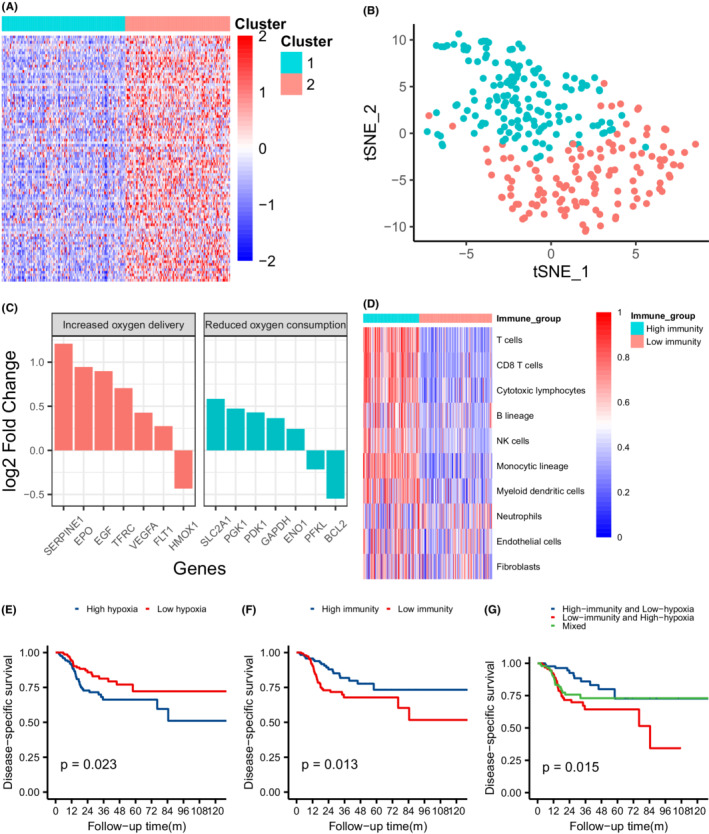
Identification of hypoxia and tumor microenvironment status and their association with response to radiotherapy in the TCGA cohort. (A) Heatmap showing the expression pattern of hypoxia gene sets in two clusters. (B) t‐SNE plots of all patients separated into two clusters labeled by color. (C) Expression changes of genes involved in the *HIF1* KEGG pathway (high hypoxia group vs. low hypoxia group). (D) Heatmap showing quantification of eight immune and two stromal populations estimated by MCP‐counter. (E) Kaplan–Meier curves of disease‐specific survival for patients in two hypoxia groups. (F) Kaplan–Meier curves of disease‐specific survival for patients in two immunity groups. (G) Kaplan–Meier curves of disease‐specific survival for patients in three hypoxia‐immunity groups

A total of 274 primary tumor samples from eligible patients in the TCGA cohort were included in the analysis. To infer the hypoxia status of the TCGA cohort, we first clustered the samples into two groups according to the Euclidian distance of their expression patterns in the “WINTER” hypoxia gene sets. Subsequently, 148 and 126 patients were clustered into cluster 1 and 2, respectively (Figure 2A). The expression level of the “WINTER” hypoxia gene sets was significantly upregulated in cluster 2 than in cluster 1. Since these gene sets consisted of upregulated genes associated with hypoxia in HNSCC, cluster 1 and 2 were annotated as the “low hypoxia” and “high hypoxia” groups, respectively. We also generated a t‐SNE plot colored by different clusters to visualize the relevance of the clusters, which separated them into two discrete groups (Figure 2B). In addition, we further analyzed the expression changes of the 26 genes involved in the *HIF1* signaling pathway, of which 14 genes were differentially expressed between the two groups (Figure 2C). Six genes (85.7%) associated with increased oxygen delivery and five genes (71.4%) associated with reduced oxygen consumption were upregulated in the “high hypoxia” group in response to hypoxia. The differences in the expression of genes associated with the *HIF1* pathway further validates the hypoxia status. The “low hypoxia” group showed a significantly better DSS than the “high hypoxia” group (log‐rank test, *P* = 0.023) (Figure 2E). The differences in survival suggest that patients with lower hypoxia status responded better to radiotherapy.

The tumor microenvironment status of the patients from the TCGA cohort was inferred from the immune scores calculated by using the ESTIMATE algorithm, which estimated the degree of immune cell infiltration in tumors. According to the cutoff value of the immune score determined by maximally selected log‐rank statistics, 119 and 155 patients from the TCGA cohort were divided into “high immunity” and “low immunity” groups, respectively (Figure S1). As estimated by MCP‐counter, the “high immunity” group showed a significantly higher abundance of eight immune cell populations, namely, T cells, CD8 T cells, cytotoxic lymphocytes, B lineage, NK cells, monocytic lineage cells, and myeloid dendritic cells, while both groups shared similar distribution patterns for the abundance of neutrophils, endothelial cells and fibroblasts (Figure 2D). The Kaplan–Meier curve showed that the DSS of the “high immunity” group was better than that of the “low immunity” group (*P* = 0.013); thus, the higher immune status was associated with better response to radiotherapy (Figure 2F).

### 3.2 IHR subtypes of the TCGA cohort

By combining the tumor immune microenvironment and hypoxia status of the patients from the TCGA cohort into a two‐dimensional index, we further divided the patients into “high immunity and low hypoxia,” “low immunity and high hypoxia,” and “mixed” groups, which included 87, 94, and 93 patients, respectively. The Kaplan–Meier curve (Figure 2G) showed a statistically significant survival difference only between “high immunity and low hypoxia” and “low immunity and high hypoxia” groups (*P* = 0.003). These survival differences indicate that a combination of hypoxia and immune status can better stratify patients in terms of their response to radiotherapy.

### 3.3 Construction and validation of the IHRGPI

A total of 274 eligible patients from the TCGA cohort were included in the training set to train an IHRGPI model to predict the response to radiotherapy. The clinicopathological characteristics of the patients in the TCGA cohort are presented in Table S1. Locoregionally advanced HNSCC patients predominated in the TCGA cohorts, with the stage III‐IV HNSCC patients accounting for 76.6%. The GEO cohorts were used as the validation set. Tables S2 and S3 demonstrate the clinicopathological characteristics of the 52 and 100 patients in GEO cohort‐1 and GEO cohort‐2, respectively. The groups showed no significant differences in age and sex. The mean age (SD) was 59.18 (10.64), 60.02 (7.92) and 57.05 (11.29) years in the TCGA cohort, GEO cohort‐1, and GEO cohort‐2, respectively, demonstrating similar distributions (*p* = 0.15), while the proportion of female patients was 21.5%, 21.2%, and 28.0%, respectively, in three cohorts. Patients with locoregionally advanced HNSCC also predominated in GEO cohort‐1 and cohort‐2, accounting for 75% and 90% of the cohorts, respectively.

**FIGURE 3:**
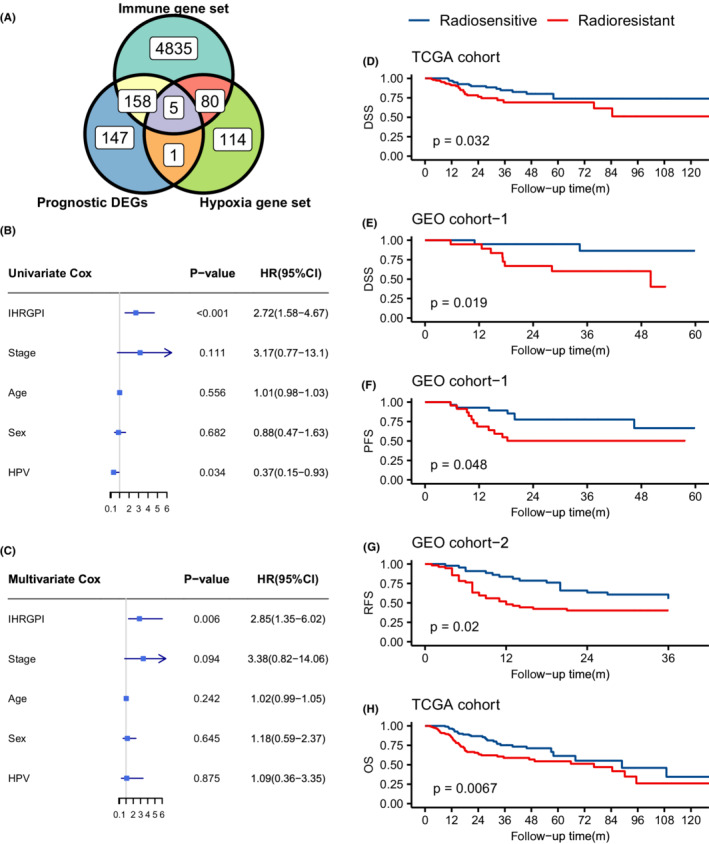
Construction and validation of the IHRGPI model. (A) Venn diagram showing the intersection between the immune‐hypoxia‐related prognostic differentially expressed genes, hypoxia gene sets, and immune gene sets. (B) Forest plot demonstrating the univariate Cox regression of IHRGPI and common clinicopathological characteristics. (C) Forest plot demonstrating the multivariate Cox regression of IHRGPI and the common clinicopathological characteristics. (D) Kaplan–Meier curves for disease‐specific survival in the TCGA cohort. (E) Kaplan–Meier curves for disease‐specific survival in GEO cohort‐1. (F) Kaplan–Meier curves for progression‐free survival in GEO cohort‐1. (G) Kaplan–Meier curves for recurrence‐free survival in GEO cohort‐2. (H) Kaplan–Meier curves for overall survival in TCGA cohort. IHRGPI, immune‐hypoxia‐related gene prognostic index; DSS, disease‐specific survival; PFS, progression‐free survival; RFS, recurrence‐free survival; OS, overall survival

A total of 2665 IHR DEGs were identified between the “high immunity and low hypoxia” and “low immunity and high hypoxia” groups from the TCGA cohort, of which 311 prognostic DEGs were significant independent prognostic factors of DSS in the univariate Cox regression. By intersecting the 324 prognostic DEGs with the hallmark hypoxia gene set from MSigDb and the immune‐related gene set from InnateDB, five IHRGs (*BCL2*, *SERPINE1*, *CAV1*, *CXCR4*, and *F3*) were finally included in the multivariate Cox regression model (Figure 3A). We constructed a model for each patient, which was calculated by the formula IHRGPI = *BCL2* × (−0.373) + *SERPINE1* × (0.225) + *CAV1* × (−0.077) + *CXCR4* × (−0.026) + *F3* × (0.113). Univariate Cox regression showed that HRGPI and HPV status were independent prognostic factors of DSS, while only IHRGPI was an independent prognostic factor in multivariate Cox regression (Figure 3B,C).

With 0 as the cutoff value for the IHRGPI, we dichotomized the patients from the TCGA cohort into the radiosensitive and radioresistant groups, which included 123 and 151 patients, respectively. After 1:1 matching, 105 patients remained in each group (Table S4). Kaplan–Meier survival analysis showed a significantly better DSS in the radiosensitive group (unmatched cohort: *p* = 0.005; matched cohort: *p* = 0.032) (Figure 3D). Univariate Cox regression showed that the hazard ratio (HR) of the radioresistant group over the radiosensitive group was 2.18 (95% CI: 1.25–3.80; *p* = 0.007) and 2.00 (95% CI: 1.05–3.79; *p* = 0.036) in the unmatched and matched TCGA cohorts, respectively. Next, we applied the IHRGPI calculation to the GEO cohort‐1with DSS as an endpoint for validation. Twenty‐eight and twenty‐four patients were divided into the radiosensitive and radioresistant groups, respectively, with 0 as the cutoff value of the IHRGPI. After 1:1 matching, 20 patients remained in each group (Table S5). Kaplan–Meier survival analysis demonstrated a better DSS in the radiosensitive group in the matched cohort (*p* = 0.019) (Figure 3E), with a corresponding HR of 5.28 (95% CI: 1.12–24.97; *p* = 0.036). Finally, the survival of the radiosensitive group remained significantly superior in the Kaplan–Meier analysis when the endpoint adopted was OS (*p* = 0.007) in the TCGA cohort, PFS (*p* = 0.048) in GEO cohort‐1, and RFS (*p* = 0.02) in GEO cohort‐2 (Figure 3F,H).

### 3.4 Hypoxic, tumor‐microenvironmental, and molecular signatures in the two IHRGPI groups

**FIGURE 4:**
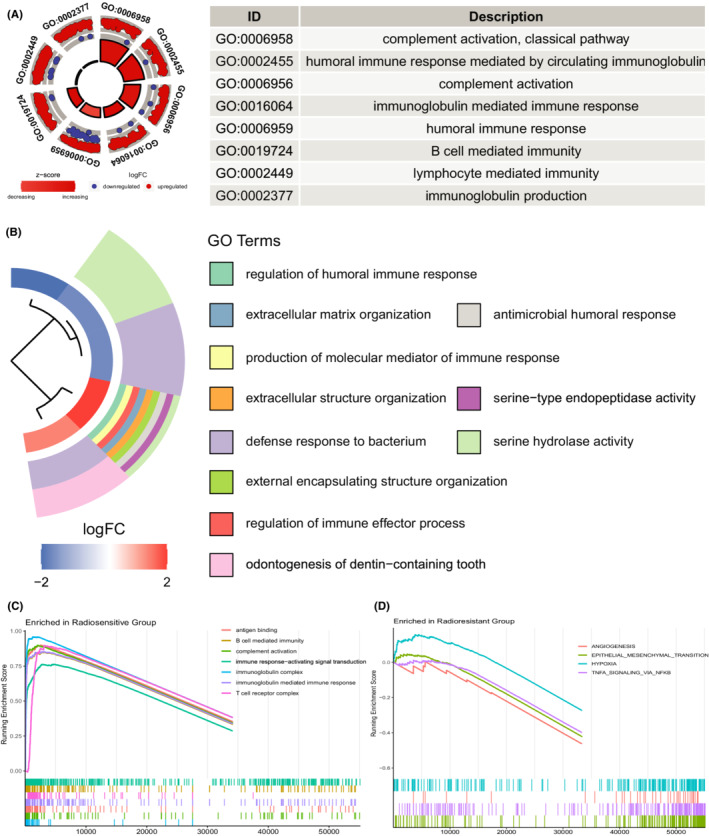
Functional enrichment analysis. (A) GOCircle plot showing the top eight GO terms based on the adjusted *p*‐values. (B) GOcluster plot showing the clustering of GO terms based on Euclidean distances and average linkage. (C) Gene sets enriched in the radiosensitive group by GSEA. (D) Gene sets enriched in the radioresistant group by GSEA. GSEA, gene set enrichment analysis

A total of 2675 radiosensitivity‐associated DEGs, including 1490 upregulated genes and 1185 downregulated genes, were identified between the radiosensitive and radioresistant groups (Figure S2). In the GO enrichment analysis (Supplementary Table), the radiosensitivity‐associated DEGs were mainly enriched in immune‐related activities, including complement activation, humoral immune response, immunoglobulin‐mediated immune response, B‐cell‐mediated immunity (Figure 4A,B). In the KEGG enrichment analysis, the DEGs were mainly enriched in cytokine‐cytokine receptor interaction, retinol metabolism and hemopoietic cell lineage (Figure S3). The association between the expression changes of each IHRG and different GO terms is demonstrated in Figure S4. Next, we performed GSEA with GO and hallmark gene sets from MSigDb. The gene sets enriched in the radiosensitive group were mainly involved in immune‐related activities, including antigen‐binding, B cell and T cell‐associated immune response, immunoglobulin‐mediated immune response, and complement activation (Figure 4C). In contrast, the hypoxia, angiogenesis, epithelial‐mesenchymal transition, and *TNFα* signaling genes via *NFκB* were mainly enriched in the radioresistant group (Figure 4D).

**FIGURE 5:**
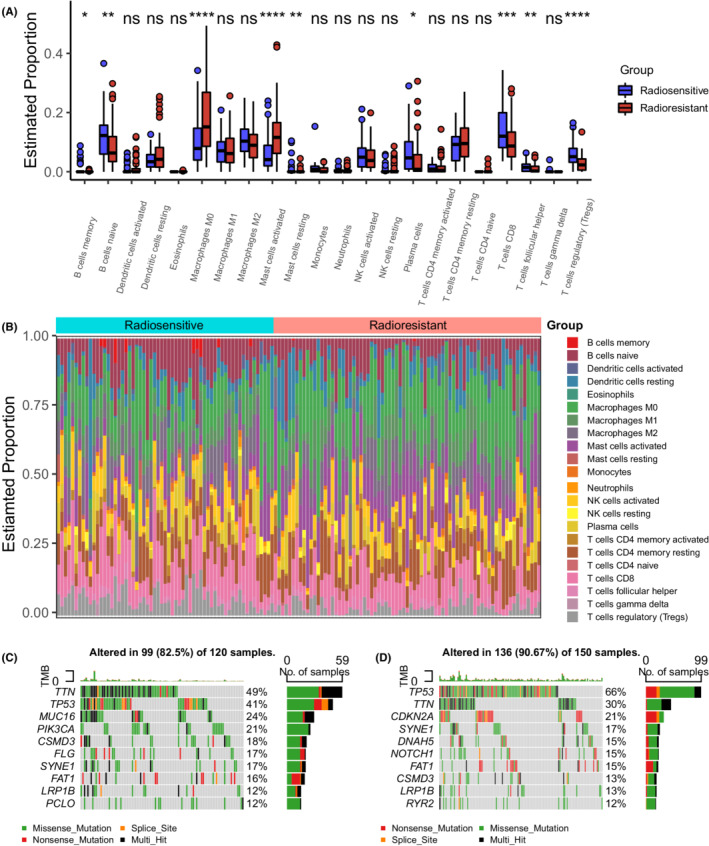
The immune and mutational landscape. (A) Boxplot comparing the proportions of different types of immune cells in tumor tissues estimated by Cibersort between the two IHRGPI groups (ns: not significant, *p < 0.05, **p < 0.01, ***p < 0.001, ****p < 0.0001). (B) 100% stacked bar plot comparing the proportion of different type of immune cells in each patient between the two IHRGPI groups. (C) Top 10 mutated genes and their mutational types in the radiosensitive group. (D) Top 10 mutated genes and their mutational types in the radioresistant group. IHRGPI, immune‐hypoxia‐related gene prognostic index

In evaluations of the intergroup differences in immune characteristics, the ESTIMATE immune score was significantly higher in the radiosensitive group (Figure S5). In analyses using Cibersort to assess the immune landscape in both groups, memory B cells, naive B cells, plasma cells, CD8 T cells, follicular helper T cells, and regulatory T cells were more abundant in the radiosensitive group, while M0 macrophages and activated mast cells were more abundant in the radioresistant group (Figure 5A,B).

We also analyzed the mutational signatures and identified the top 10 mutated genes of both groups (Figure 5C,D). Missense mutations predominated in both groups (Figure S6). *TP53*, *TTN*, *SYNE1*, and *FAT1* were present among the top 10 mutated genes of both groups. The mutation rates of *CDKB2A*, *DNAH5*, and *NOTCH1* were higher in the radioresistant group, while those of *MUC16*, *PIK3CA*, *CSMD3*, and *FLG* were higher in the radiosensitive group. The TMB did not differ significantly between the two groups (*p* = 0.33) (Figure S7). Finally, the top 10 transcriptional factors associated with the five selected IHRGs using the ChEA3 tool are listed in Table S6, including *FOXD1*, *ZNF469*, *NR3C1*, *PPARG*, *SMAD3*, *MEOX1*, *BCL6B*, *HIF1A*, *ELK3*, and *FOSL1*, of which *HIF1A* was elevated significantly in the radioresistant group in the TCGA cohort (*p* < 0.01), GEO cohort‐1 (*p* = 0.01) and GEO cohort‐2 (*p* < 0.01).

### 3.5 Treatment strategies

**FIGURE 6:**
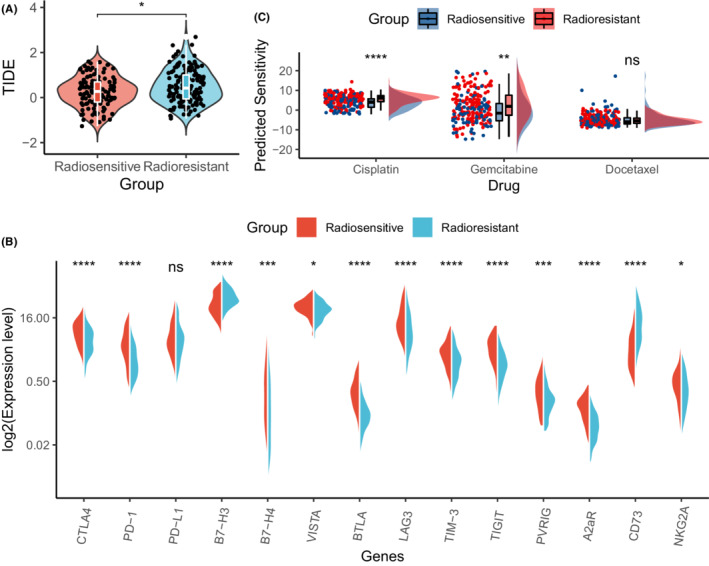
Sensitivity to different types of treatment in the two IHRGPI groups. (A) Violin plot showing the difference in TIDE score between the two IHRGPI groups. (B) Split violin plot demonstrating the difference in log‐transformed expression levels of the well‐studied targets of the immune checkpoint inhibitors between the two IHRGPI groups. (C) Rainfall plot comparing the predicted sensitivity to three commonly‐used chemotherapeutic drugs between the two IHRGPI groups. (ns: not significant, *p < 0.05, **p < 0.01, ***p < 0.001, ****p < 0.0001). TIDE, Tumor Immune Dysfunction and Exclusion; IHRGPI, immune‐hypoxia‐related gene prognostic index

To explore potentially effective combinations of radiotherapy and other treatment strategies, we calculated the TIDE score for each patient. The TIDE score was significantly lower in the radiosensitive group (*p* = 0.019), indicating that a better response to radiotherapy correlates with a better response to immunotherapy (Figure 6A). Moreover, the proportion of predicted responders to immune checkpoint inhibitors was higher in the radiosensitive group (37.4%) than in the radioresistant group (27.2%). However, there was no statistically significant intergroup difference in the TMB. As for the differences in the expression levels of the targets of well‐studied immune checkpoint inhibitors (Figure 6B), the levels of *CTLA4*, *PD1*, *B7H4*, *VISTA*, *BTLA*, *LAG3*, *TIM3*, *TIGIT*, *PVRIG*, *A2aR* and *NKG2A* were significantly higher in the radiosensitive group, while the levels of *B7H3* and *CD73* were higher in the radioresistant group. With the “pRRophetic” algorithm,^29^ we estimated the sensitivity to three common chemotherapeutic drugs in both groups. The radioresistant group showed a significantly higher sensitivity to gemcitabine and cisplatin, while no statistically significant difference was detected in the sensitivity to docetaxel (Figure 6C).

## 4 DISCUSSION

Radiotherapy is one of the three major treatment modalities for HNSCC, and establishing models to predict patients' radiotherapy response can facilitate individualized treatment decisions. Although several predictive models have been reported,^6^, ^7^, ^8^, ^9^ most of them were purely statistically driven without a radiobiological foundation, so they were difficult to explain. Intrinsic radiosensitivity is not the only determinant factor of the response to radiotherapy^5^; the tumor immune microenvironment and hypoxia also play important roles in this process. Reoxygenation and reactivation of the immune system are among the well‐known traditional “5 Rs” and the newly proposed “6th R”, respectively. Thus, we developed the IHRGPI to predict HNSCC patients' response to radiotherapy based on the tumor immune microenvironment and hypoxia signature by using the transcriptomic data in the TCGA database. The IHRGPI was proven to be a valid predictor of the radiosensitivity of HNSCC, showing better survival in the radiosensitive group after treatment with radiotherapy in both TCGA and GEO cohorts.

Five IHRGs were included in the IHRGPI. Among them, *BCL2* is an anti‐apoptotic protein. However, previous studies on the effect of *BCL2* on have yielded contradictory findings,^30^, ^31^, ^32^ precluding its validation as a reliable biomarker of prognosis or radiosensitivity. Theoretically, increased expression of *BCL2*, an apoptosis suppressor gene, will block apoptosis and lead to resistance to chemoradiotherapy. However, higher *BCL2* expression was instead associated with a better response to radiotherapy in this study. These contradictory findings can be attributed to multiple mechanisms. Because apoptosis is regulated by a complex network, pro‐apoptotic signals should be taken into consideration while interpreting these findings. In our study, the ratio of *BCL2* to *BAX*, a well‐known pro‐apoptotic gene, was not significantly different between the two IHRGPI groups in the TCGA cohort (*p* = 0.99), indicating a balance between pro‐apoptotic and anti‐apoptotic signals. On the other hand, *BCL2* overexpression was reported to block DNA double‐strand break repair via nonhomologous end‐joining and enhance radiosensitivity.^31^, ^33^ *SERPINE1* also plays an important role in the *HIF1* pathway by increasing oxygen delivery in response to hypoxia,^34^ and was also included in previously proposed gene signatures to predict locoregional tumor control for HNSCC patients treated with postoperative radiotherapy^35^ or the response to neo‐adjuvant chemoradiotherapy before surgery for locally advanced esophageal cancer.^36^ *CAV1* encodes the main component of the lipid domains on plasma membranes known as caveolae. Although it promoted radioresistance in cancer cell lines,^37^ loss of its stromal expression was also reported to mediate the resistance of prostate cancer to radiotherapy.^38^ In this study, upregulated *CAV1* was associated with a better response to radiotherapy. Since bulk RNA sequencing of HNSCC tissue provides the average gene expression of both tumor cells and stromal cells, our result may mainly reflect the influence of stromal *CAV1* expression on the radiosensitivity of HNSCC, and methodologies with higher resolution may better elucidate our findings. *CXCR4* is a specific chemokine receptor for stromal cell‐derived factor‐1 that is expressed on cancer cells and many other cells in the tumor microenvironment, and has many important functions including promoting tumor progression and metastasis and facilitating angiogenesis.^39^ Although post‐irradiation *CXCR4*‐mediated angiogenesis is associated with radioresistance and recurrence after the radiotherapy through rescuing the damaged tumor vasculature caused by radiation,^40^, ^41^ upregulated *CXCR4* was associated with better response to radiotherapy in this study. *CXCR4*‐mediated angiogenesis may improve the hypoxia status of the tumor in the pre‐treatment sample and thereby sensitize the cancer cells to radiation. *F3* encodes coagulation factor III, a cell‐surface glycoprotein that can initiate blood coagulation cascades in the extrinsic coagulation pathway, and has also been reported to participate in tumor angiogenesis,^42^, ^43^ but its association with radioresistance remains to be elucidated.

For all five genes, *HIF1A* was one of the top 10 transcriptional factors in the transcription factor enrichment analysis by ChEA3. *HIF1A* is a master regulator of the cellular and systematic response to hypoxia.^44^ *HIF1A* expression was elevated significantly in the radioresistant group in the three cohorts. GSEA also showed that hypoxia and angiogenesis were mainly enriched in the radioresistant group.

The complex interactions between and synergistic effects of radiotherapy and anticancer immunity have been extensively explored and elucidated,^45^ and reactivation of the immune system was lately proposed as the “6th R"^5^ In this study, patients in “high immunity” group showed better response to radiotherapy than those in “low immunity” group. Thus, our results further confirmed the role of immunity in radiotherapy. Many immune‐related activities, including complement activation and humoral immune response, were also significantly enriched between the two IHRGPI groups in both GO and GSEA functional enrichment analysis, and CD8 T cells, the most powerful effector cells in the anticancer immune response,^46^ were more abundant in the tumor microenvironment of HNSCC patients in both the “high immunity” and radiosensitive groups in comparison with the “low immunity” and radioresistant groups, respectively. Taken together, these findings indicated that anticancer immunity did play an important role in HNSCC patients' response to radiotherapy, and an immune‐active tumor microenvironment favored a better response to radiotherapy among HNSCC patients.

Advances in radiotherapy techniques, such as the advent of IMRT, have facilitated significantly better dose distribution and constraint. However, they did not significantly improve prognosis in most studies.^2^ Therefore, we sought to identify an optimal combinational treatment strategy for patients with different radiosensitivity. Our results suggest that radioresistant patients might benefit more from chemotherapy, while radiosensitive patients might benefit more from immunotherapy. Therefore, for patients who receive radiotherapy and have lower IHRGPI, immunotherapy could be recommended, and chemotherapy might be omitted or de‐intensified, while chemotherapy could not be omitted in those with high IHRGPI. Notably, although the TIDE scores suggested that the radiosensitive group generally responded better to the immunotherapy, the expression levels of *B7H3* and *CD73* were higher in the radioresistant group, indicating that the radioresistant group might benefit more from *B7H3* and *CD73* inhibitors.

This study had several limitations. First, we did not evaluate the effectiveness of radiotherapy in the two IHRGPI groups among all patients, and we only evaluated the prognostic significance of the IHRGPI groups among all patients receiving radiotherapy in each cohort. For non‐metastatic HNSCC, surgery remains the mainstay of treatment strategies, while radiotherapy is usually adjuvant therapy for high‐risk postoperative patients. Therefore, patients receiving radiotherapy usually bear more factors indicating a poor prognosis. Because the indications for adjuvant radiotherapy are complicated, and information regarding the rationale for treatment decisions is insufficient, evaluating the effectiveness of radiotherapy in two IHRGPI groups among all patients respectively will introduce more confounding factors that could not be adjusted. Second, we did not explore the effectiveness of the combination of radiotherapy and targeted therapy, since that reliable biomarkers for the response to cetuximab, an epithelial growth factor receptor inhibitor commonly used in treating HNSCC, are not yet been available.

In conclusion, we developed and validated the IHRGPI in HNSCC patients, which was based on the tumor microenvironment and hypoxia signature and can facilitate prediction of the response to radiotherapy. We also explored the potential mechanisms underlying the differences between two IHRGPI groups. Moreover, our model provides individualized suggestions for combinational treatment strategies involving different IHRGPI groups.

## AUTHOR CONTRIBUTIONS

GLZ, KBY, and CX have made contributions to the design of the work, the acquisition and analysis of data, drafting and revision of the work. RJF and WFL have made contributions to the design and revision of the work. JM have made contributions to the design and revision of the work, supervision of the work and funding acquisition. All authors read and approved the final manuscript.

## CONFLICT OF INTEREST

The authors declare that they have no competing interests.

## ETHICS STATEMENT

The requirements of patients' informed consent and institutional review board approval were waived given the datasets were de‐identified and publicly available.

## Supporting information


Figure S1
Figure S2
Figure S3
Figure S4
Figure S5
Figure S6
Figure S7

Table S1
Table S2
Table S3
Table S4
Table S5
Table S6

## Data Availability

The data analyzed in this study were obtained from the Cancer Genome Atlas (TCGA) at TCGA‐HNSC and Gene Expression Omnibus (GEO) at GSE117973 and GSE39366.
