# Supplementary material for: Development of a prediction model for radiotherapy response among patients with head and neck squamous cell carcinoma based on the tumor immune microenvironment and hypoxia signature
Source: Cancer Med. 2022 May 3;11(23):4673–87. doi: 10.1002/cam4.4791 (PMC9741991; doi:10.1002/cam4.4791)
Supplement: Supplementary file 1 — Figure S1 Figure S2 Figure S3 Figure S4 Figure S5 Figure S6 Figure S7 [file CAM4-11-4673-s002.docx]

Supplementary Figures

**Supplementary Figure 1**. Distribution of the immune score and the standardized log-rank statistics for selection of the optimal cutoff immune score.

**Supplementary Figure 2**. Volcano plot of differentially expressed genes between the two IHRGPI groups (radiosensitive versus radioresistant group).

**Supplementary Figure 3**. Bar plot of the KEGG enrichment analysis of differentially expressed genes between the two IHRGPI groups.

IHRGPI, immune hypoxia–related gene prognostic index.

**Supplementary Figure 4**. GOChord plot demonstrating the correlation between the expression changes in genes involved in the IHRGPI and the GO terms.

IHRGPI, immune hypoxia–related gene prognostic index.

**Supplementary Figure 5**. Comparison of the ESTIMATE immune score between the two IHRGPI groups.

IHRGPI, immune hypoxia–related gene prognostic index.

**Supplementary Figure 6**. Mutational characteristics in the two IHRGPI groups. (A) Radioresistant group; (B) radiosensitive group.

IHRGPI, immune hypoxia–related gene prognostic index.

**Supplementary Figure 7**. Comparison of TMB between the two IHRGPI groups.

TMB, tumor mutational burden; IHRGPI, immune hypoxia–related gene prognostic index.


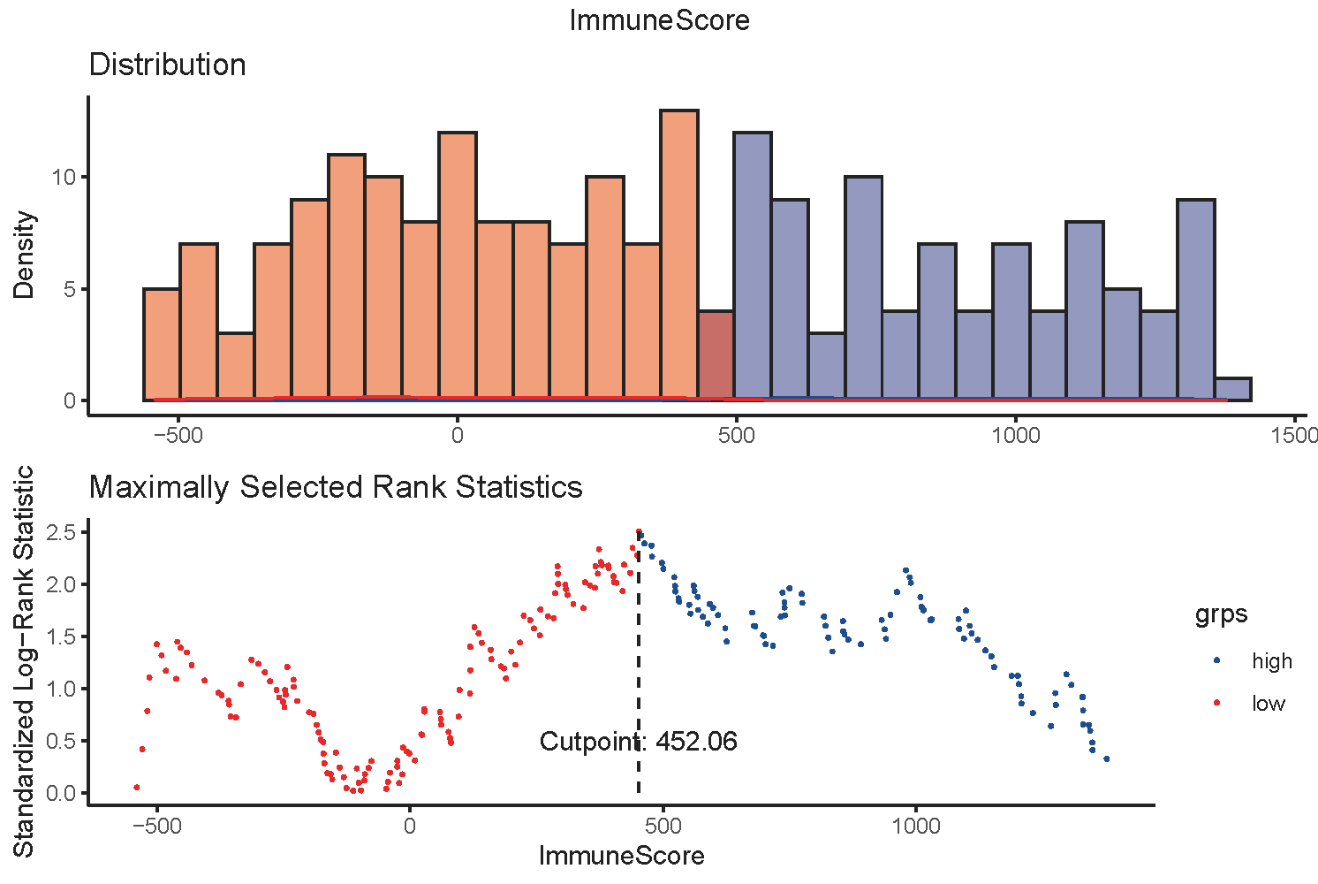
**Supplementary Figure 1**. Distribution of the immune score and the standardized log-rank statistics for selection of the optimal cutoff immune score.


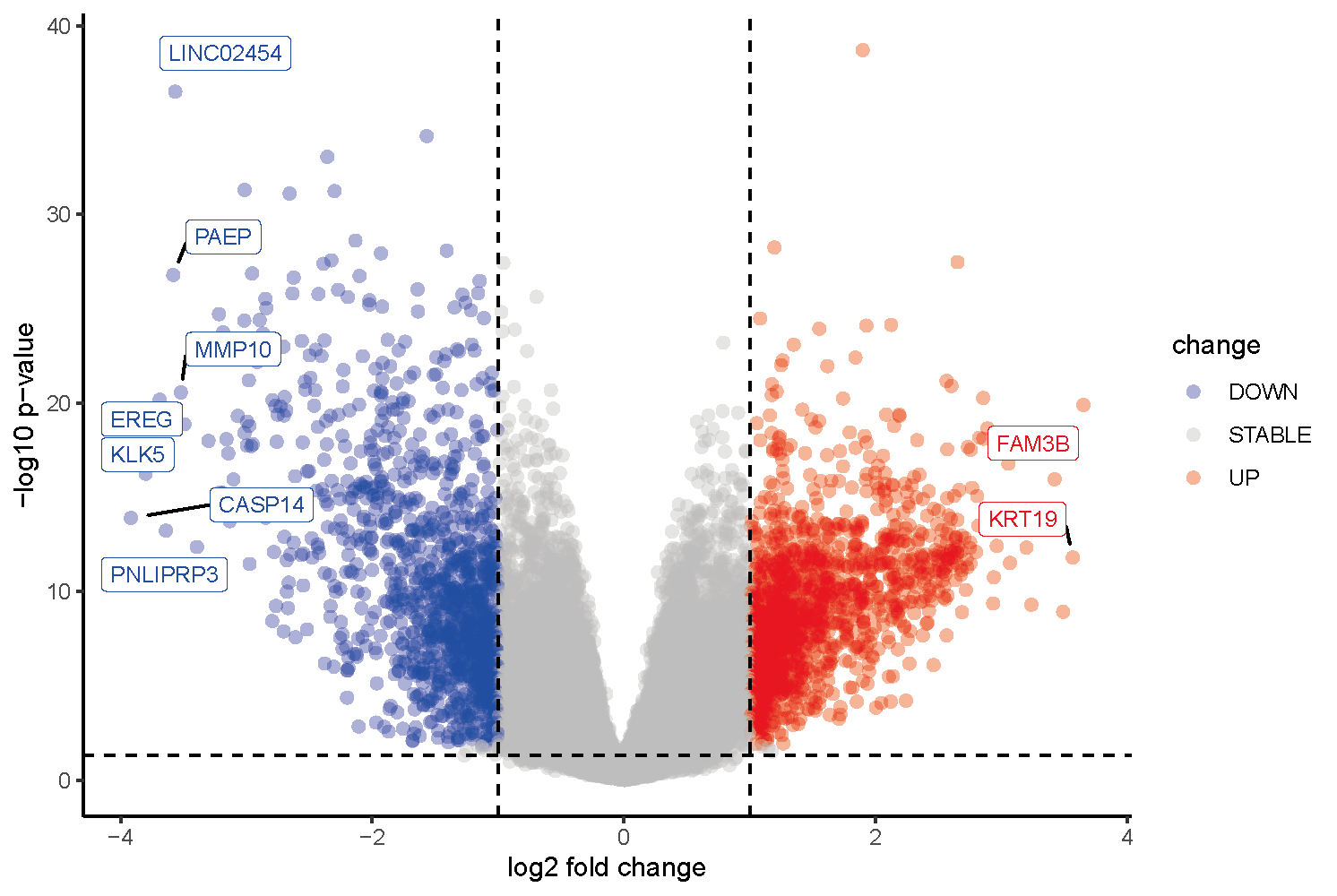
**Supplementary Figure 2**. Volcano plot of differentially expressed genes between the two IHRGPI groups (radiosensitive versus radioresistant group).

**
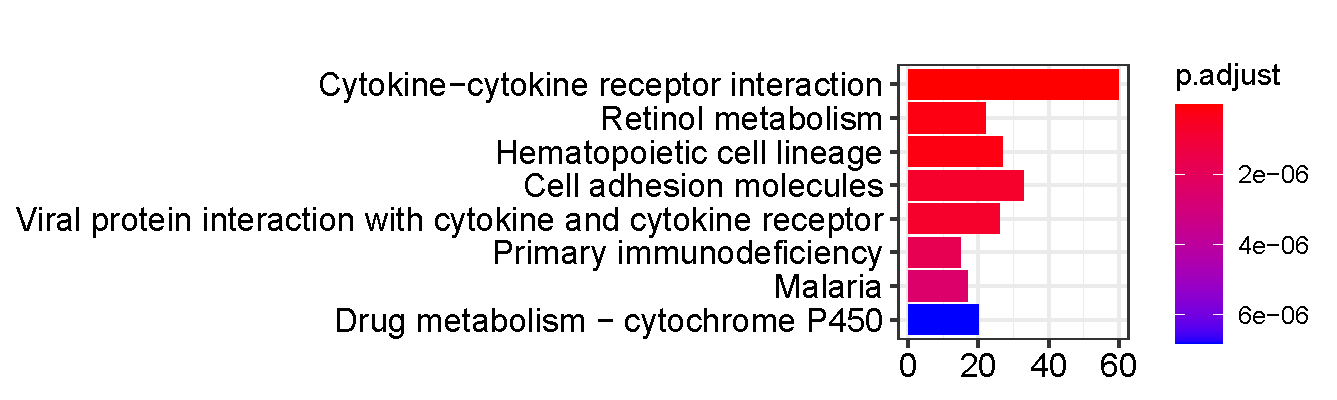
**

**Supplementary Figure 3**. Bar plot of the KEGG enrichment analysis of differentially expressed genes between the two IHRGPI groups.

IHRGPI, immune hypoxia–related gene prognostic index.

**
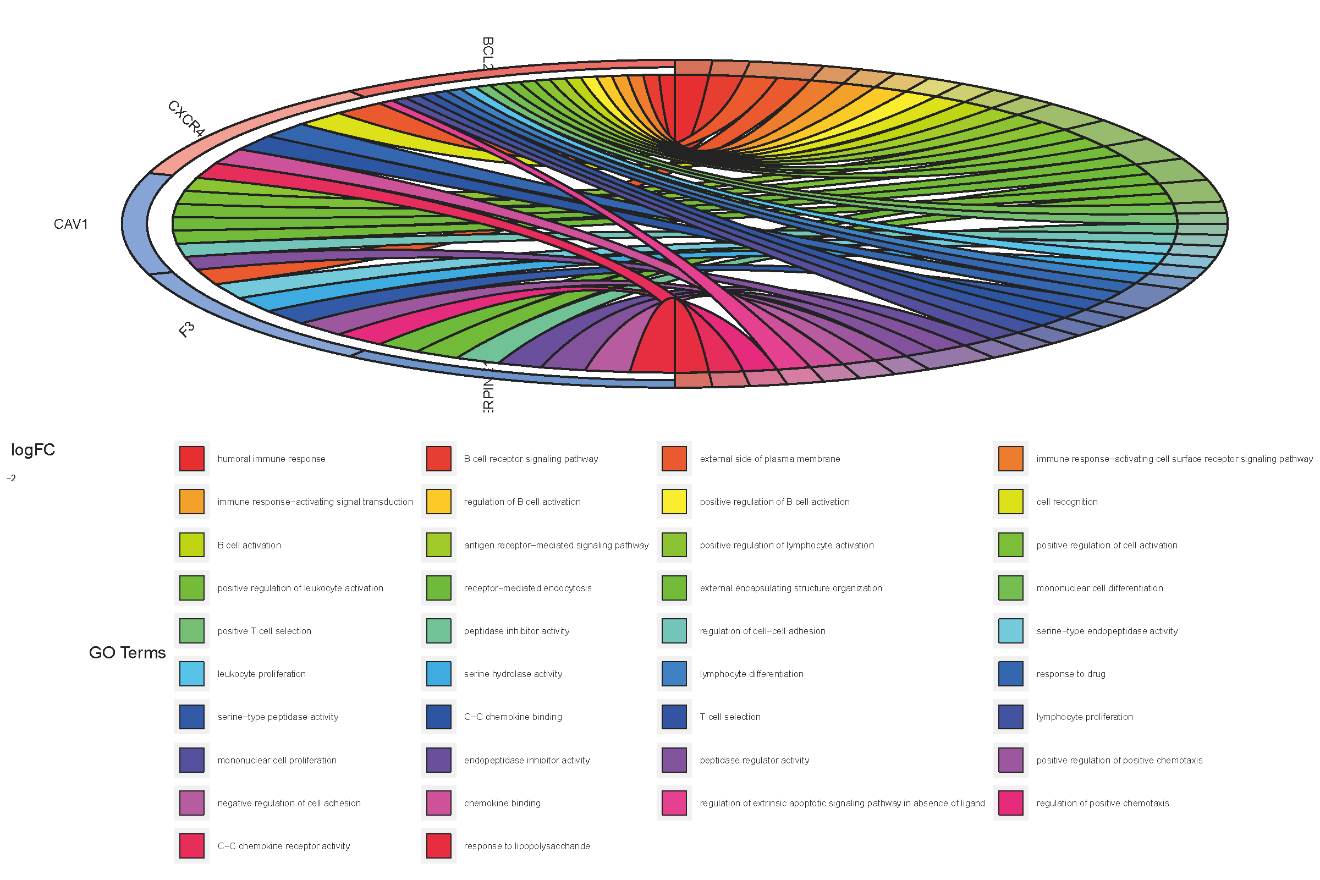
Supplementary Figure 4**. GOChord plot demonstrating the correlation between the expression changes in genes involved in the IHRGPI and the GO terms.

IHRGPI, immune hypoxia–related gene prognostic index.

**
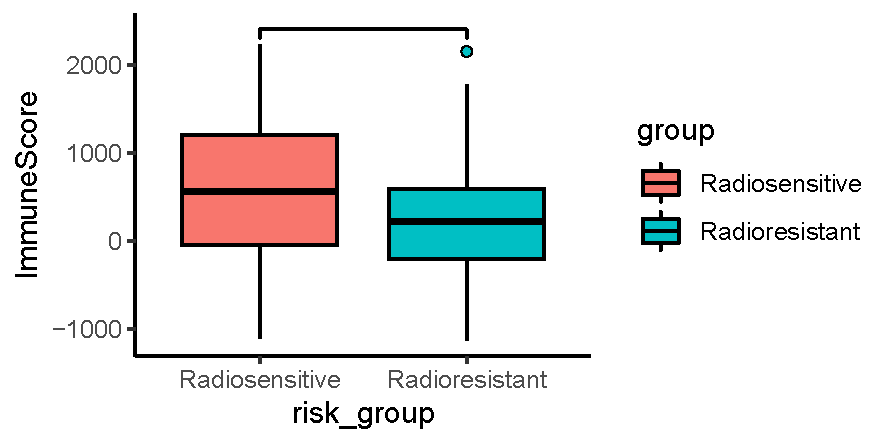
Supplementary Figure 5**. Comparison of the ESTIMATE immune score between the two IHRGPI groups.

IHRGPI, immune hypoxia–related gene prognostic index.

**
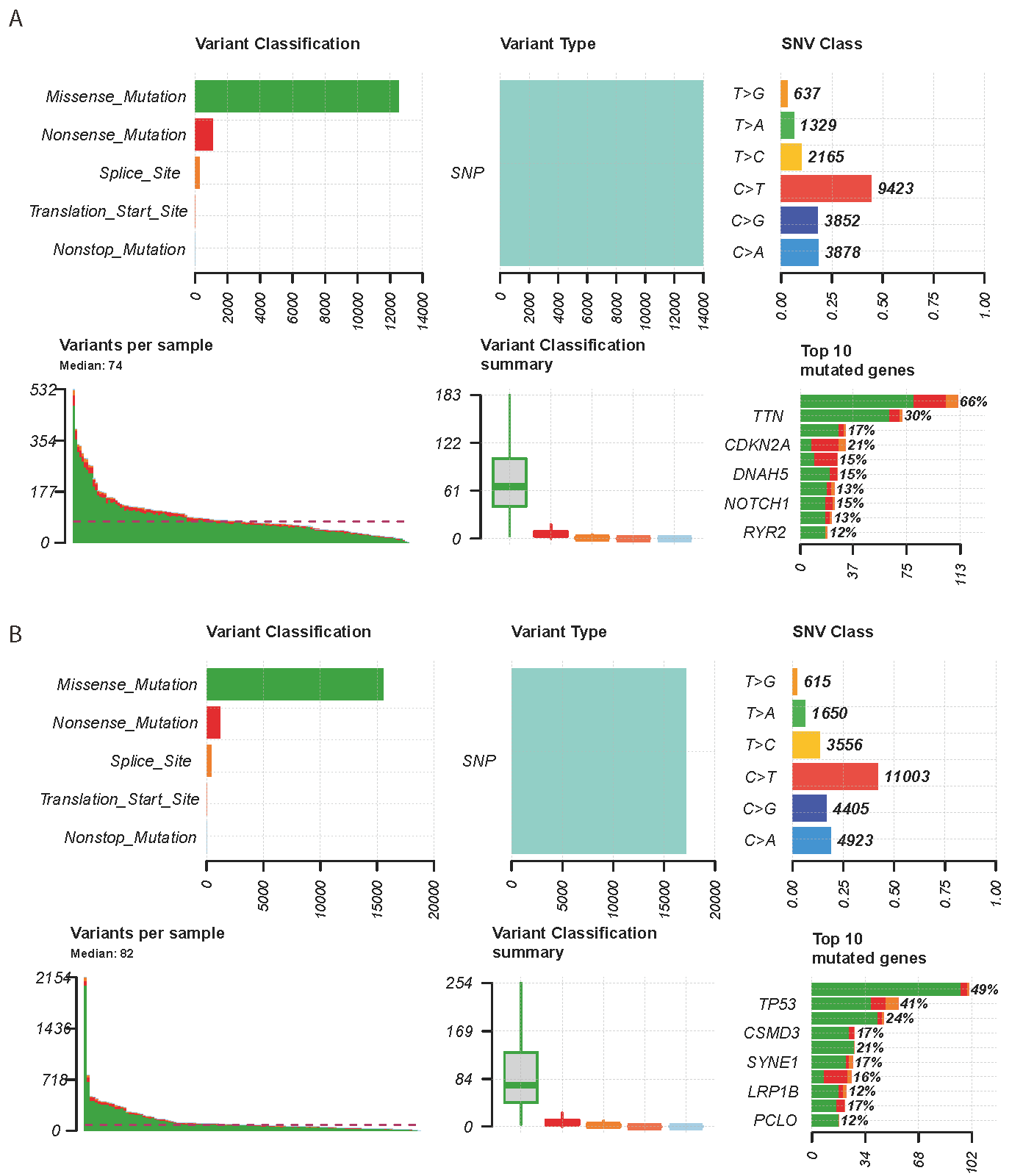
**

**Supplementary Figure 6**. Mutational characteristics in the two IHRGPI groups. (A) Radioresistant group; (B) radiosensitive group.

IHRGPI, immune hypoxia–related gene prognostic index.

**
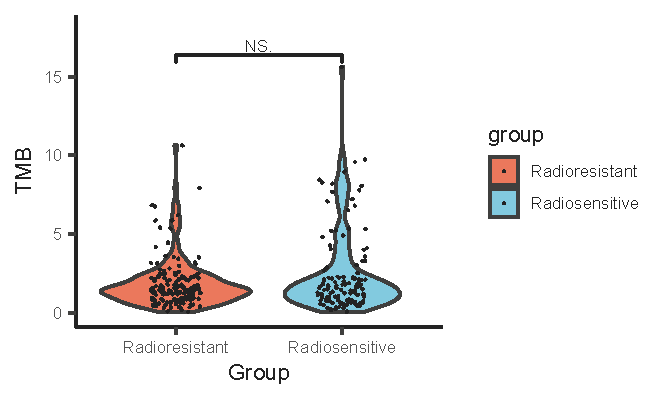
**

**Supplementary Figure 7**. Comparison of TMB between the two IHRGPI groups.

TMB, tumor mutational burden; IHRGPI, immune hypoxia–related gene prognostic index.
