# Supplementary material for: Development of a prediction model for radiotherapy response among patients with head and neck squamous cell carcinoma based on the tumor immune microenvironment and hypoxia signature
Source: Cancer Med. 2022 May 3;11(23):4673–87. doi: 10.1002/cam4.4791 (PMC9741991; doi:10.1002/cam4.4791)
Supplement: Supplementary file 2 — Table S1 Table S2 Table S3 Table S4 Table S5 Table S6 [file CAM4-11-4673-s001.docx]

# Table S1. Clinicopathological characteristics of patients from the TCGA cohort

|  | **Overall**  **(n = 274)** |
| --- | --- |
| Age (mean (SD)) | 59.18 (10.64) |
| Sex (%) |  |
| Female | 59 (21.5) |
| Male | 215 (78.5) |
| HPV (%) |  |
| - | 221 (80.7) |
| + | 48 (17.5) |
| Unknown | 5 (1.8) |
| AJCC T category (%) |  |
| T1 | 12 (4.4) |
| T2 | 55 (20.1) |
| T3 | 74 (27.0) |
| T4 | 125 (45.6) |
| TX | 6 (2.2) |
| Unknown | 2 (0.7) |
| AJCC N category (%) |  |
| N0 | 106 (38.7) |
| N1 | 49 (17.9) |
| N2 | 102 (37.2) |
| N3 | 4 (1.5) |
| NX | 11 (4.0) |
| Unknown | 2 (0.7) |

| AJCC overall stage (%) |  |
| --- | --- |
| I | 7 (2.6) |
| II | 15 (5.5) |
| III | 36 (13.1) |
| IVA | 168 (61.3) |
| IVB | 6 (2.2) |
| Unknown | 42 (15.3) |

| Alcohol history (%) |  |
| --- | --- |
| - | 74 (27.0) |
| + | 197 (71.9) |
| Unknown | 3 (1.1) |

# Table S2. Clinicopathological characteristics of patients from GEO cohort-1.

|  | **Overall**  **(n = 52)** |
| --- | --- |
| Age (mean (SD)) | 60.02 (7.92) |
| Sex |  |
| Female | 11 (21.2) |
| Male | 41 (78.8) |
| HPV (%) |  |
| - | 34 (65.4) |
| + | 18 (34.6) |
| T category (%) |  |
| T1 | 5 (9.6) |
| T2 | 19 (36.5) |
| T3 | 12 (23.1) |
| T4 | 16 (30.8) |
| N category (%) |  |
| N0 | 16 (30.8) |
| N1 | 13 (25.0) |
| N2 | 23 (44.2) |
| Alcohol history |  |
| - | 25 (48.1) |
| + | 27 (51.9) |

# Table S3. Clinicopathological characteristics of patients from GEO cohort-2.

|  | | **Overall**  **(n = 100)** | | |
| --- | --- | --- | --- | --- |
| Age (mean (SD)) | | 57.05 (11.29) | | |
| Sex (%) |  | | | |
| Female | | 28 (28.0) | | |
| Male | | 72 (72.0) | | |
| HPV (%) | |  | | |
| - | | 60 (60.0) | | |
| + | | 12 (12.0) | | |
| Unknown | | 28 (28.0) | | |
| Stage (%) | |  |  |  |
| I | | 3 (3.0) | | |
| II | | 5 (5.0) | | |
| III | | 20 (20.0) | | |
| IV | | 70 (70.0) | | |
| Unknown | | 2 (2.0) | | |
| T category (%) |  | | | |
| T1 | | 7 (7.0) | | |
| T2 | | 16 (16.0) | | |
| T3 | | 19 (19.0) | | |
| T4 | | 41 (41.0) | | |
| Unknown | | 17 (17.0) | | |
| N category (%) |  | | | |
| N0 | | 24 (24.0) | | |
| N1 | | 14 (14.0) | | |
| N2 | | 40 (40.0) | | |
| N3 | | 5 (5.0) | | |
| Unknown | | 18 (18.0) | | |
| Alcohol (%) |  | | | |
| Heavy | | 41 (41.0) | | |
| None or Light/Moderate | | 59 (59.0) | | |

**Table S4. Tumor and nodal category distribution in the TCGA cohort before and after 1:1 propensity score matching**

|  | **Unmatched** | | | **Matched** | | |
| --- | --- | --- | --- | --- | --- | --- |
|  | Better response | Worse response | P-value | Better response | Worse response | P-value |
| n | 123 | 151 |  | 105 | 105 |  |
| T category (%) |  |  | 0.006 |  |  | 1.000 |
| T1/2 | 41 (33.3) | 26 (17.2) |  | 25 (23.8) | 25 (23.8) |  |
| T2/3/4 | 78 (63.4) | 121 (80.1) |  | 78 (74.3) | 78 (74.3) |  |
| TX | 4 (3.3) | 2 (1.3) |  | 2 (1.9) | 2 (1.9) |  |
| Not reported | 0 (0.0) | 2 (1.3) |  | 0 (0.0) | 0 (0.0) |  |
| N category (%) |  |  | 0.598 |  |  | 0.143 |
| N0 | 46 (37.4) | 60 (39.7) |  | 46 (43.8) | 60 (57.1) |  |
| N1/2/3 | 72 (58.5) | 85 (55.0) |  | 55 (52.4) | 41 (39.0) |  |
| NX | 5 (4.1) | 6 (4.0) |  | 4 (3.8) | 4 (3.8) |  |
| Not reported | 0 (0.0) | 2 (1.3) |  | 0 (0.0) | 0 (0.0) |  |

**Table S5. Tumor and nodal category distribution in the GEO cohorts before and after 1:1 propensity score matching**

|  | **Unmatched** | | | **Matched** | | |
| --- | --- | --- | --- | --- | --- | --- |
|  | Better response | Worse response | P-value | Better response | Worse response | P-value |
| n | 28 | 24 |  | 20 | 20 |  |
| T category (%) |  |  | 0.150 |  |  | 1.000 |
| T1/2 | 16 (57.1) | 8 (33.3) |  | 8 (40.0) | 8 (40.0) |  |
| T3/4 | 12 (429) | 16 (66.7) |  | 12 (60.0) | 12 (60.0) |  |
| N category (%) |  |  | 0.945 |  |  | 0.301 |
| N0 | 8 (28.6) | 8 (33.3) |  | 8 (33.3) | 4 (20.3) |  |
| N1/2 | 20 (71.4) | 16 (66.7) |  | 12 (66.7) | 16 (80.0) |  |

**Table S6. Top 10 transcription factors associated with the selected immune-hypoxia genes by using the ChIP-X Enrichment Analysis 3 tool**

| Rank | Transcription factor | Score | Library | Overlapping_Genes |
| --- | --- | --- | --- | --- |
| 1 | FOXD1 | 5.5 | ARCHS4 Coexpression,10;GTEx Coexpression,1 | CAV1,SERPINE1 |
| 2 | ZNF469 | 26.5 | ARCHS4 Coexpression,47;GTEx Coexpression,6 | SERPINE1 |
| 3 | NR3C1 | 36.5 | Literature ChIP-seq,50;ARCHS4 Coexpression,104;ENCODE ChIP-seq,17;Enrichr Queries,20;ReMap ChIP-seq,4;GTEx Coexpression,24 | CAV1,SERPINE1,BCL2,CXCR4,F3 |
| 4 | PPARG | 40.8 | Literature ChIP-seq,24;ARCHS4 Coexpression,43;Enrichr Queries,53;ReMap ChIP-seq,71;GTEx Coexpression,13 | CAV1,SERPINE1,BCL2,CXCR4,F3 |
| 5 | SMAD3 | 41 | Literature ChIP-seq,9;ARCHS4 Coexpression,57;Enrichr Queries,30;ReMap ChIP-seq,22;GTEx Coexpression,87 | CAV1,SERPINE1,BCL2,CXCR4,F3 |
| 6 | MEOX1 | 44 | ARCHS4 Coexpression,29;Enrichr Queries,70;GTEx Coexpression,33 | CAV1,SERPINE1,CXCR4 |
| 7 | BCL6B | 47.33 | ARCHS4 Coexpression,1;Enrichr Queries,101;GTEx Coexpression,40 | CAV1,SERPINE1,CXCR4 |
| 8 | HIF1A | 50.2 | Literature ChIP-seq,27;ARCHS4 Coexpression,70;Enrichr Queries,49;ReMap ChIP-seq,86;GTEx Coexpression,19 | CAV1,SERPINE1,BCL2,CXCR4 |
| 9 | ELK3 | 54.25 | Literature ChIP-seq,39;ARCHS4 Coexpression,24;Enrichr Queries,147;GTEx Coexpression,7 | CAV1,SERPINE1,CXCR4 |
| 10 | FOSL1 | 61 | ARCHS4 Coexpression,74;ENCODE ChIP-seq,14;Enrichr Queries,162;ReMap ChIP-seq,23;GTEx Coexpression,32 | CAV1,SERPINE1,CXCR4,F3 |
